# Protein-Interaction Affinity Gradient Drives [4Fe–4S] Cluster Insertion in Human Lipoyl Synthase

**DOI:** 10.1021/jacs.1c13626

**Published:** 2022-03-28

**Authors:** Giovanni Saudino, Simone Ciofi-Baffoni, Lucia Banci

**Affiliations:** † Magnetic Resonance Center (CERM), University of Florence, Via L. Sacconi 6, 50019 Sesto Fiorentino, Italy; ‡ Department of Chemistry “Ugo Schiff”, University of Florence, Via della Lastruccia 3, 50019 Sesto Fiorentino, Italy; # Consorzio Interuniversitario Risonanze Magnetiche di Metalloproteine (CIRMMP), Via L. Sacconi 6, 50019 Sesto Fiorentino, Italy

## Abstract

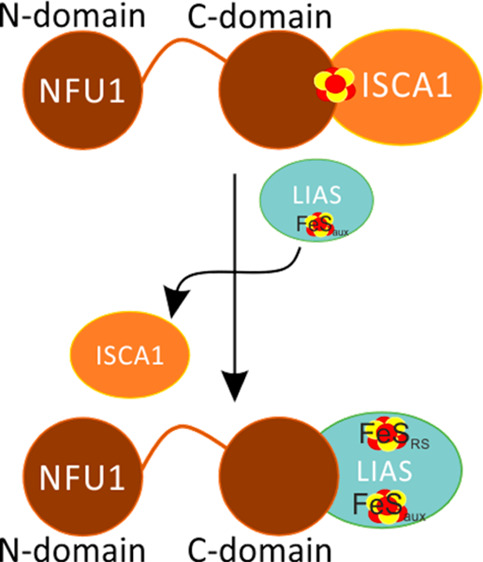

Human lipoyl synthase (LIAS) is an enzyme containing two [4Fe–4S] clusters (named FeS_RS_ and FeS_aux_) involved in the biosynthesis of the lipoyl cofactor. The mechanism by which a [4Fe–4S] cluster is inserted into LIAS has thus far remained elusive. Here we show that NFU1 and ISCA1 of the mitochondrial iron–sulfur cluster assembly machinery, via forming a heterodimeric complex, are the key factors for the insertion of a [4Fe–4S] cluster into the FeS_RS_ site of LIAS. In this process, the crucial actor is the C-domain of NFU1, which, by exploiting a protein-interaction affinity gradient increasing from ISCA1 to LIAS, drives the cluster to its final destination.

Human lipoyl synthase (LIAS) is a member of the radical *S*-adenosylmethionine (SAM) superfamily and catalyzes the final step of the biosynthesis of lipoyl cofactor.^1,2^ LIAS binds two [4Fe–4S] clusters:^3,4^ a [4Fe–4S] cluster (FeS_RS_), typical of all radical SAM enzymes,^5^ and a [4Fe–4S] cluster (FeS_aux_) that provides two sulfur atoms to the lipoyl cofactor.^6^ LIAS interacts with human NFU1, a member of the mitochondrial iron–sulfur cluster (ISC) assembly machinery.^7,8^ NFU1 is required for LIAS cluster maturation.^9−11^ However, to date no direct information are available on whether and how human NFU1 inserts a [4Fe–4S] cluster into LIAS. Our present data show the key molecular factors that drive the insertion of a [4Fe–4S] cluster into the FeS_RS_ site of LIAS.

As the first step, we have investigated, by nuclear magnetic resonance (NMR) and analytical gel filtration, the interaction between apo NFU1 and as-isolated LIAS (AI LIAS, hereafter), which contains a [4Fe–4S]^2+^ cluster bound mostly at the FeS_aux_ site (see the Experimental Section in the Supporting Information for details, Table S1 and Figure S1). In the analytical gel filtration chromatogram of a 1:1 apo NFU1–AI LIAS mixture, a main peak containing both proteins is present with an elution volume smaller than that of the two isolated proteins (Figure S2). The elution volume of this peak is consistent with the presence of a heterodimeric complex, which is the predominant form at the 1:1 apo NFU1–AI LIAS ratio. We also observed that, when apo ^15^N-NFU1 is stepwise titrated with AI LIAS up to a 1:1 protein ratio, chemical shift changes occurred in the ^1^H-^15^N heteronuclear single quantum coherence (HSQC) maps of apo NFU1 in intermediate/slow exchange regimes on the NMR time scale, indicating the occurrence of the apo NFU1–AI LIAS interaction (Figures 1A and S3). The majority of the affected residues are located in the C-domain of NFU1 (see Figures S3 and S4 for details), thus revealing the C-domain of NFU1 as the crucial player driving the NFU1–AI LIAS interaction. These data are in agreement with previous yeast-two-hybrid assay studies.^8^

**Figure 1:**
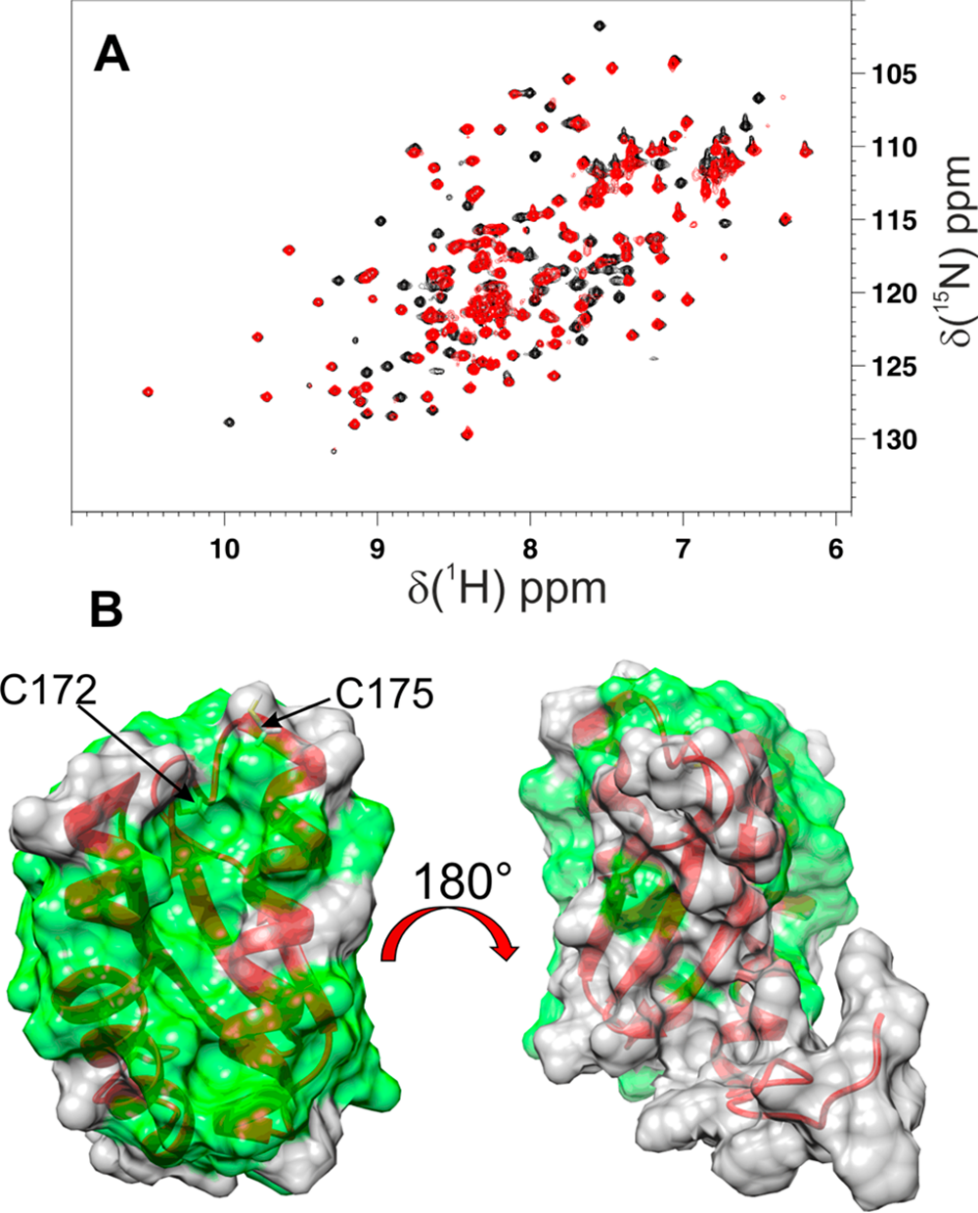
Apo NFU1 interacts with AI LIAS via its C-terminal domain. (A) Overlay of ^1^H-^15^N HSQC maps of ^15^N-apo NFU1 (black) and the 1:1 ^15^N-apo NFU1-unlabeled AI LIAS mixture (red). (B) Meaningful chemical shift changes are shown in green on the structure of the C-domain of apo NFU1.

By mapping these changes on the structure of the C-terminal domain of apo NFU1,^12^ we observed that the two helices of the C-terminal domain of apo NFU1 are significantly affected by the protein–protein interaction, while the β-sheet is essentially unaffected (Figure 1B). The cluster binding CXXC motif of apo NFU1, encased between the two helices, is also involved in the interaction with AI LIAS, indicating that AI LIAS in the complex with NFU1 is positioned close to the cluster-binding region.

Complex formation was also followed by performing in parallel ^1^H-^15^N HSQC spectra and analytical gel filtrations on protein mixtures obtained by adding one or more equivalents of ^15^N-apo NFU1 to unlabeled AI LIAS (see the Supporting Information for details). At a 1:1 protein ratio, apo NFU1 is fully complexed with AI LIAS, as no signal of isolated apo NFU1 is present in the NMR spectrum (Figure 2). Analytical gel filtration chromatogram of this 1:1 mixture showed the peak of the heterodimeric complex with a tail that covers the elution volumes of both isolated monomeric apo NFU1 and AI LIAS, indicating that a low portion of the heterodimeric complex dissociates upon the dilution effect of the gel filtration (Figures 2 and S2). Upon addition of two equivalents of apo NFU1, the NMR signal of isolated apo NFU1 was observed (Figure 2). This result rules out the formation of a heterotrimeric complex composed by two molecules of NFU1 and one molecule of AI LIAS. This model is confirmed by the analytical gel filtration performed on the same mixture, which retains the peak of the heterodimeric complex and additionally showed an increase of the intensity of the peaks corresponding to the monomeric and dimeric isolated apo NFU1 (Figure 2). Upon addition of three and four equivalents of apo NFU1, the NMR signal of isolated apo NFU1 increases in intensity and concomitantly, in the chromatogram of the analytical gel filtration, the peaks of monomeric and dimeric isolated apo NFU1 gradually increase their intensity with respect to the peak of the heterodimeric complex (Figure 2). In fraction 2 of the sodium dodecyl-sulfate polyacrylamide gel electrophoresis (SDS-PAGE) (Figure 2), we can consistently observe the increase of the intensity of the NFU1 band with respect to that of AI LIAS along the additions of apo NFU1.

**Figure 2:**
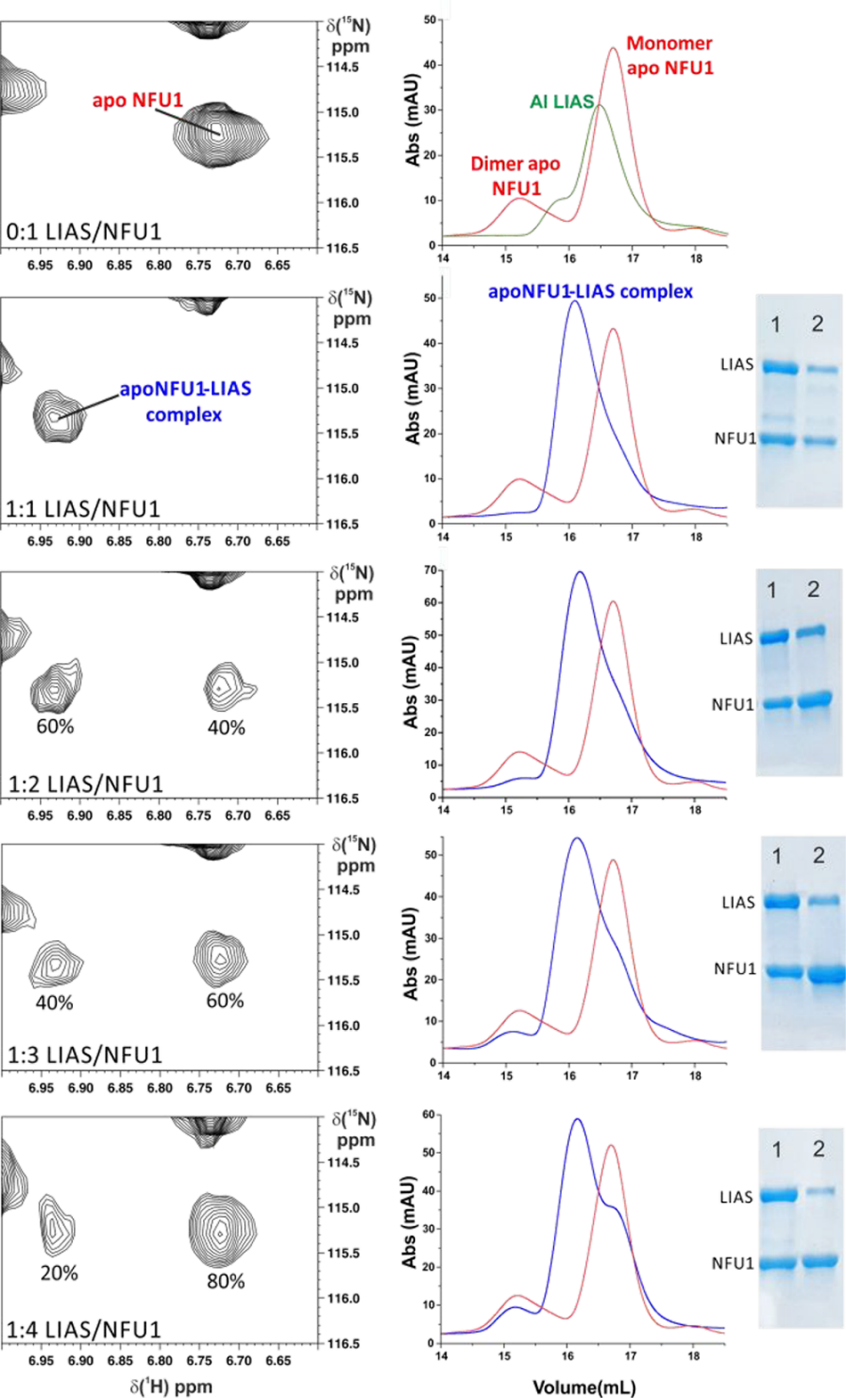
Apo NFU1 and AI LIAS form a heterodimeric complex. On the left, ^1^H-^15^N HSQC maps at different NFU1–AI LIAS ratios enclosing the signal of Arg 96 of ^15^N-NFU1 in slow exchange regime on the NMR time scale upon NFU1–AI LIAS complex formation. On the right, analytical gel filtration chromatograms of the same mixtures analyzed by NMR. SDS-PAGE of fraction 1 (eluted between 16.0 and 16.5 mL) and that of fraction 2 (eluted at 16.5–17.0 mL) are reported on the right of each chromatogram.

As the following step, unlabeled AI LIAS was stepwise added to the apo ISCA1–^15^N-NFU1 complex obtained as previously described.^13^ The overlay of the ^1^H-^15^N HSQC maps of the two individual apo unlabeled ISCA1–^15^N-NFU1 and apo ^15^N-NFU1–unlabeled AI LIAS complexes clearly shows that the spectra of these two complexes are different (Figure S5) and thus they can be exploited to monitor a possible conversion between the two complexes. Upon addition of one equivalent of AI LIAS, several NMR signals of ^15^N-NFU1 complexed with apo ISCA1 broaden beyond detection or change their chemical shifts (Figure 3A), indicating that apo NFU1 changes its interactions pattern. When this spectrum is compared with that of the heterodimeric complex between apo ^15^N-NFU1 and unlabeled AI LIAS (Figure 3B), it results that the two spectra are well superimposable, indicating that apo NFU1 is preferentially interacting with AI LIAS to form the apo NFU1–AI LIAS heterodimeric complex. Consistently, no NMR signals of free apo ^15^N-NFU1 are observed (compare black spectrum in Figure 3A with green spectrum in Figure 3B), indicating that NFU1 remains in a complexed form. The analytical gel filtration of the final mixture showed an intense peak with an elution volume smaller than those of the three isolated monomeric proteins and of the heterodimeric ISCA1–NFU1 complex (Figure 3C and Figure S2), consistent with the formation of the higher molecular weight apo NFU1–AI LIAS dimeric complex. Furthermore, a low-intensity peak eluting at 17.7 mL is formed upon addition of AI LIAS to the apo ISCA1–NFU1 complex (Figure 3C) whose elution volume matches with that of monomeric apo ISCA1, thus indicating that ISCA1 is released in solution as a free protein. In conclusion, NMR and analytical gel filtration data allow to exclude the formation of a heterotrimeric ISCA1-NFU1-AI LIAS complex and show that AI LIAS displaces ISCA1 from the heterodimeric apo ISCA1-NFU1 complex to form a heterodimeric complex with NFU1.

**Figure 3:**
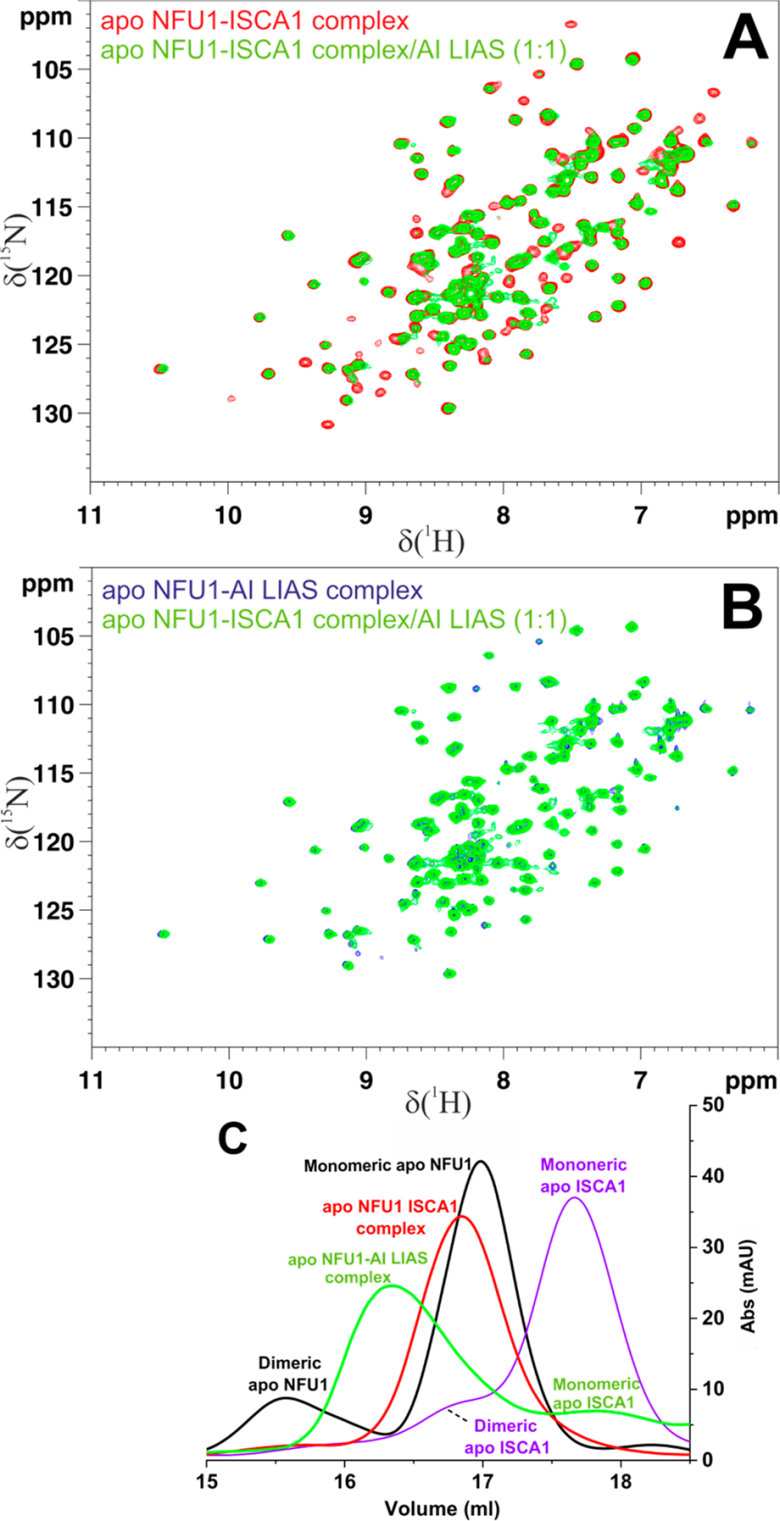
AI LIAS displaces ISCA1 from the apo ISCA1–NFU1 complex to form a heterodimeric complex with apo NFU1. (A,B) Overlay of ^1^H-^15^N HSQC maps of ^15^N-apo NFU1 in different states indicated by color codes. (C) Analytical gel filtration chromatograms of apo ISCA1 (violet), apo NFU1 (black), apo ISCA1–NFU1 complex (red), and a 1:1 mixture between apo ISCA1–NFU1 complex and AI LIAS (green).

The [4Fe–4S] cluster insertion into the FeS_RS_ site of AI LIAS was then investigated. Previous findings^13,14^ support the model that the ISCA1–NFU1 complex is a suitable physiological candidate, although it may not be the only possibility,^15,16^ to insert a [4Fe–4S]^2+^ cluster into LIAS. ^1^H-^15^N HSQC experiments titrating the [4Fe–4S]^2+^ unlabeled ISCA1–^15^N-NFU1 complex with unlabeled AI LIAS up to a 1:1 ratio were then performed. Some NMR signals allowed us to monitor the cluster release from complexed ^15^N-NFU1, as their chemical shifts exclusively depend on the presence ([4Fe–4S] in Figure 4A,B) or absence (apo in Figure 4A,B) of the [4Fe–4S]^2+^ cluster in NFU1 complexed with either unlabeled AI LIAS or ISCA1 (Figures S5 and S6). In the final mixture of the titration, these signals of complexed ^15^N-NFU1 overlay with those corresponding to the formation of apo complexed ^15^N-NFU1 (Figure 4A,B), thus indicating that the [4Fe–4S]^2+^ cluster is no longer bound to NFU1. The ^1^H-^15^N HSQC spectra also showed that the signals of the final 1:1 mixture overlap with those of the apo ^15^N-NFU1–AI LIAS complex and not with those of the apo ISCA1–^15^N-NFU1 complex (Figure S7), indicating the formation of the apo state of NFU1 complexed with LIAS. Thus, the displacement of ISCA1 from the [4Fe–4S]^2+^ ISCA1–NFU1 complex to form a dimeric complex between NFU1 and LIAS occurs similarly to what was observed in the absence of cluster transfer (Figure 3).

**Figure 4:**
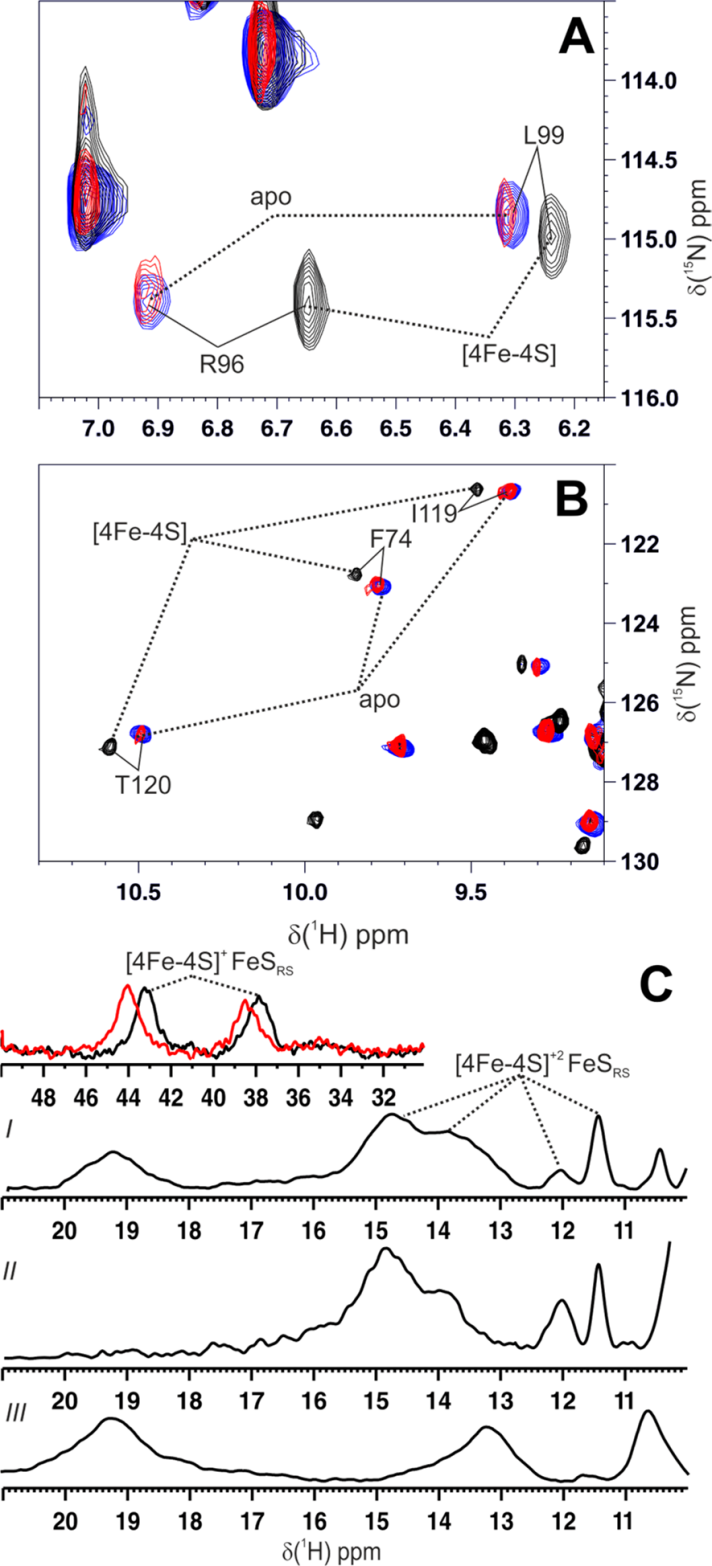
[4Fe–4S]^2+^ ISCA1–NFU1 transfers the cluster to the FeS_RS_ site of AI LIAS. (A and B) Overlay of two different regions of ^1^H-^15^N HSQC maps of [4Fe–4S]^2+^ ISCA1–NFU1 complex (black), apo NFU1–AI LIAS complex (blue) and of a 1:1 mixture of the [4Fe–4S]^2+^ ISCA1–NFU1 complex and AI LIAS (red) (NFU1 is ^15^N-labeled, ISCA1 and AI LIAS are unlabeled). (C) Paramagnetic 1D ^1^H NMR spectra of (*I*) a 1:1 mixture of [4Fe–4S]^2+^ ISCA1–NFU1 and C106/C111/C117A AI LIAS, obtained by anaerobically mixing the two proteins, (*II*) C106/C111/C117A AI LIAS and (*III*) . In the inset of panel *I*, a far-shifted region of the paramagnetic NMR spectrum is shown at 298 K (black) and 290 K (red).

We also followed cluster insertion into LIAS by paramagnetic 1D ^1^H NMR. A triple C106/C111/C117A LIAS variant was used as cluster acceptor because this variant lacks the cysteine ligands of FeS_aux_ and can allow exclusively monitoring cluster insertion into FeS_RS_. C106/C111/C117A AI LIAS was purified with ∼30% of [4Fe–4S] clusters bound to the FeS_RS_ site (Table S1). Upon addition of one equivalent of the [4Fe–4S]^2+^ ISCA1–NFU1 complex to the C106/C111/C117A AI LIAS variant, the intensities of the ^1^H NMR signals at 15–11 ppm, assigned to βCH_2_ of the ligands of FeS_RS_, increase in intensity (Figure 4C), indicating that cluster insertion into the FeS_RS_ site occurred, thus being the cluster not degraded or released in solution.

The anti-Curie temperature dependence and the chemical shift values of these signals are consistent with an oxidized [4Fe–4S]^2+^ cluster bound to LIAS, in agreement with the UV–visible spectrum of the final 1:1 mixture (Figure S8). In addition, we observed two other signals in the 46–36 ppm region with Curie temperature dependence (inset of Figure 4C*I*). Their temperature dependence and chemical shifts are typical of protons of cysteine residues bound to a reduced [4Fe–4S]^+^ cluster,^17^ thus indicating that a fraction of FeS_RS_ is in the reduced state. The presence of cysteine residues with chemical shifts typical of both reduced and oxidized [4Fe–4S] clusters suggests that FeS_RS_ can be partially reduced by 5 mM dithiothreitol (DTT), the only reductant present in the mixture. This result is in agreement with what previously observed in wild-type LIAS,^3^ is fully consistent with the electron transfer function of FeS_RS_ in the catalytic mechanism^18^ as well as with a reduction potential of FeS_RS_ lower than that of DTT, as typically observed for radical SAM [4Fe–4S] clusters.^19^

In conclusion, we have shown that the C-domain of NFU1 is the trigging factor for the insertion of a [4Fe–4S] cluster into the FeS_RS_ site of LIAS thanks to its specific interaction with LIAS. The strength of this interaction displaces ISCA1 complexed with NFU1 via the competition for the same binding site, which consists of the two packed helices of the C-domain of NFU1. Thus, the C-domain of NFU1 results a stronger interacting partner of LIAS than ISCA1. In the mitochondrial ISC assembly machinery, the C-domain drives first [4Fe–4S]^2+^ cluster delivery from the ISCA1–ISCA2 complex, where the [4Fe–4S]^2+^ cluster is assembled,^14^ to the [4Fe–4S]^2+^ ISCA1–NFU1 intermediate complex,^13^ which then specifically directs the cluster into the FeS_RS_ site of LIAS. These sequential molecular events are driven by an interaction affinity gradient of the C-domain of NFU1 increasing from ISCA1 to LIAS. Our data do not exclude that a dimeric [4Fe–4S]^2+^ NFU1-dependent pathway might be present in human cells, as proposed in yeast,^15^ as an alternative pathway.

## Supporting Information Available

Detailed experimental procedures (PDF)
