# Supplementary material for: Protein-Interaction Affinity Gradient Drives [4Fe–4S] Cluster Insertion in Human Lipoyl Synthase
Source: J Am Chem Soc. 2022 Mar 28;144(13):5713–7. doi: 10.1021/jacs.1c13626 (PMC8991016; doi:10.1021/jacs.1c13626)
Supplement: Supplementary file 1 — ja1c13626_si_001.pdf [file ja1c13626_si_001.pdf]

Supporting Information

A protein-interaction affinity gradient drives [4Fe-4S] cluster insertion in human lipoyl synthase

Giovanni Saudino,† Simone Ciofi-Baffoni,*,†,‡ and Lucia Banci*,†,‡,#

†Magnetic Resonance Center (CERM), University of Florence, Via L. Sacconi 6, 50019 Sesto Fiorentino, Italy.

‡Department of Chemistry "Ugo Schiff", University of Florence, Via della Lastruccia 3, 50019 Sesto Fiorentino, Italy.

#Consorzio Interuniversitario Risonanze Magnetiche di Metalloproteine (CIRMMP), Via L. Sacconi 6, 50019 Sesto Fiorentino, Italy.

This PDF file includes:

Experimental Section
Tables S1
Figures S1 to S8
References

Experimental Section

Protein production

Human NFU1 and human ISCA1 were expressed and purified as previously described.[1-2] Following these procedures, NFU1 was isolated in the apo form and ISCA1 was obtained largely in the apo form, with ~30% in a $[2Fe\text{-}2S]^{2+}$ cluster form.[2]

Human lipoyl synthase (LIAS) and two triple C106/C111/C117A and C137/C141/C144 LIAS variants were produced following the same protocol reported in Camponeschi *et al.*[3] with the exception that no $FeCl_3$ was added in the culture at the time of induction in order to decrease the Fe-S cluster content. Following this procedure, wild-type LIAS was isolated with one bound [4Fe-4S] cluster (**Table S1**, named as-isolated (AI) hereafter) out of the two [4Fe-4S] clusters typically bound to the FeS_{aux} and FeS_{RS} sites of lipoyl synthases.[3,4] This result fully reproduces what it has been recently reported for LIAS using the same expression conditions, i.e. with no addition of $FeCl_3$ at the time of induction.[5] In order to define which FeS cluster site (i.e. FeS_{aux} vs. FeS_{RS}) is occupied in AI LIAS, we performed paramagnetic 1D ^1H NMR spectrum of the wild-type LIAS protein produced in this study and compared it with that of wild-type LIAS that contains [4Fe-4S] clusters on both FeS_{aux} and FeS_{RS} sites. The latter was obtained using a high iron content in the expression medium as already described in the literature.[3] It results that only the NMR signals of protons bound to the FeS_{aux} site are detected in LIAS isolated from the low iron content cell culture, supporting that the occupancy of the FeS_{RS} site by a [4Fe-4S] cluster is below 10% (see for details **Figure S1**). This result is also supported by the production of two triple C106/C111/C117A and C137/C141/C144 LIAS variants obtained in the same low iron content expression conditions used for the wild-type protein. By following the same anaerobic purification protocol used for the wild-type protein, both mutants were isolated with a [4Fe-4S] cluster (**Figure S8**), as reported by Camponeschi *et al.*.[3] The triple C137/C141/C144 LIAS variant, which lacks the cysteine ligands of the FeS_{RS} cluster and can thus bind only the FeS_{aux} cluster, has ~90% of [4Fe-4S] cluster site occupancy (**Table S1**), while the C106/C111/C117A LIAS variant, which can bind, on the contrary, only the FeS_{RS} cluster, has a ~30% of [4Fe-4S] cluster site occupancy (**Table S1**). These results suggest that the applied protein expression conditions (i.e. low iron content) largely favours an occupancy of the FeS_{aux} cluster site with respect to that of the FeS_{RS} cluster site. Thus, they support that the estimated 50% of [4Fe-4S] cluster site occupancy found for wild-type AI LIAS results from a [4Fe-4S] cluster bound to Aux site. Our mutagenesis data nicely fit with those recently published showing that the C137A LIAS variant, in which the FeS_{RS} cluster binding was abolished, binds a [4Fe-4S] cluster with full cluster occupancy to the FeS_{aux} site, while the C106A LIAS variant, in which the FeS_{aux} cluster binding was abolished, shows no evidence for a [4Fe-4S] bound cluster, once the variant was aerobically purified.[5] In conclusion, both our data and literature data[5] showed

that the FeS_{aux} cluster-binding site is preferentially occupied once wild-type Al LIAS is produced in Luria-Bertani media with no supplemental iron source provided.

The $[4Fe\text{-}4S]^{2+}$ cluster bound form of the ISCA1-NFU1 complex was obtained by chemical reconstitution and spectroscopically characterized as already reported in the literature.[2] Non-heme iron and acid-labile sulfide content on the as-isolated proteins was determined as previously described.[6] Protein concentration was determined by use of the Bradford assay.

Analytical size exclusion chromatography

Analytical size exclusion chromatography was performed on purified samples with a Superdex 200 Increase 10/300 GL column attached to an AKTA pure chromatography unit using a continuous flow rate of 0.6 mL/min. The column was calibrated with gel filtration marker calibration kit, 6500-66000 Da (Sigma-Aldrich), to obtain the apparent molecular masses of the detected species. The column was equilibrated with nitrogen-purged 50 mM phosphate buffer at pH 7.0 containing 150 mM NaCl and 5 mM DTT. Each experiment was successfully repeated at least three times.

NMR spectroscopy

^{1}H-^{15}N HSQC experiments were performed at 298 K in nitrogen-purged 50 mM phosphate buffer, 150 mM NaCl, 5 mM DTT pH 7.0, 10% (v/v) D_2O. These NMR spectra were recorded on Bruker AVANCE 900 and 950 MHz, processed using the standard Bruker software (Topspin) and analyzed with CARA program.[7]

In order to monitor protein-protein interaction between Al LIAS and apo NFU1, NMR titration experiments were performed in anaerobic conditions (additions performed in glove-box) adding unlabeled Al LIAS to the NMR tube containing ^{15}N-labeled apo NFU1 (0.2-0.3 mM) up to a 1:1 ratio as well as adding one to four equivalents of ^{15}N-labelled apo NFU1 to the NMR tube containing unlabeled Al LIAS (0.2-0.3 mM). The experimental set up of the last titration allow us to better exploit the backbone NH signal of Arg 96, which significantly change its chemical shifts in a slow exchange regime on the NMR time scale, to monitor the possible formation of a heterotrimeric complex composed by two molecules of NFU1 and one molecule of Al LIAS. Indeed, in the case of the formation of a ternary complex, the backbone NH signal of Arg 96 in the uncomplexed apo NFU1 form would not appear at a 1:2 Al LIAS:apo NFU1 ratio in our experimental conditions, as, on the contrary, it occurs as shown in Figure 2 of the main text. The interaction between Al LIAS and apo NFU1 complexed with ISCA1 was followed titrating, in anaerobic conditions (additions performed in glove-box), unlabeled ISCA1-^{15}N-labeled apo NFU1 complex, prepared as previously described,[2] with unlabeled Al LIAS. Cluster transfer between chemically reconstituted $[4Fe\text{-}4S]^{2+}$ ISCA1-NFU1 and Al LIAS was followed titrating, in anaerobic conditions (additions performed in

glove-box), $[4Fe-4S]^{2+}$ unlabeled ISCA1-^{15}N labelled NFU1 complex with unlabeled Al LIAS (either wild type or C106/C111/C117A Al LIAS variant) up to a 1:1 ratio. Each titration was successfully repeated three times.

All NMR titration data were analyzed comparing the ^{1}H-^{15}N HSQC spectra recorded along the additions of the protein partner with that of the initial state as well as with ^{1}H-^{15}N HSQC spectra of the appropriate proteins and complexes in their apo and holo forms. Following the chemical shift changes observed in the ^{1}H-^{15}N HSQC maps along each stepwise titration we were able to assign the residues affected by protein-protein interaction. The observed chemical shift changes were reported as backbone weighted average chemical shift differences, i.e. $\Delta\delta_{avg}(HN)$, i.e. $(((\Delta H)^2 + (\Delta N/5)^2)/2)^{1/2}$, where ΔH and ΔN are chemical shift differences for backbone amide ^{1}H and ^{15}N nuclei, respectively. Signals showing both broadening beyond detection effects and chemical shift changes were considered to map the interaction surface on NFU1 upon its interaction with Al LIAS. A threshold value, obtained by averaging $\Delta_{avg}(HN)$ values plus one standard deviation (1σ), was used to define chemical shift differences meaningful when they are higher than 1σ plus $\Delta_{avg}(HN)$ ppm, following the standard procedure used in NMR protein-protein interaction studies.[8] Chemical shift assignment of full-length apo NFU1 was available in the Biological Magnetic Resonance Bank (under accession codes BMRB: 26801).[9]

1D ^{1}H paramagnetic NMR experiments were acquired at 400 MHz with a ^{1}H optimized 5 mm probe at temperatures ranging from 283 K and 298 K, with protein samples in nitrogen-purged 50 mM phosphate buffer, 150 mM NaCl, 5 mM DTT pH 7.0, 99% or 10% (v/v) D_2O. Protein concentration was in the range of 0.4-0.6 mM. Water signal was suppressed via fast repetition experiments and water selective irradiation.[10] Experiments were typically performed using an overall recycle delay of 60 ms. Squared cosine and exponential multiplications were applied prior to Fourier transformation. Manual baseline correction was performed, using polynomial functions. Each experiment was successfully repeated three times.

Table S1. Iron and acid-labile sulfide quantification of as-isolated (AI) LIAS proteins.

Sample	Fe[a]	S[a]	[4Fe-4S] cluster site occupancy
Wild-type LIAS	3.9 ± 0.1	4.1 ± 0.1	50%
C137/C141/C144 LIAS variant	3.3 ± 0.1	3.9 ± 0.1	90%
C106/C111/C117A LIAS variant	1.2 ± 0.2	1.3 ± 0.2	30%

[a]Fe and acid-labile S measurements are reported as mol Fe or S per mol of monomeric protein. Data are the average of three independent samples.

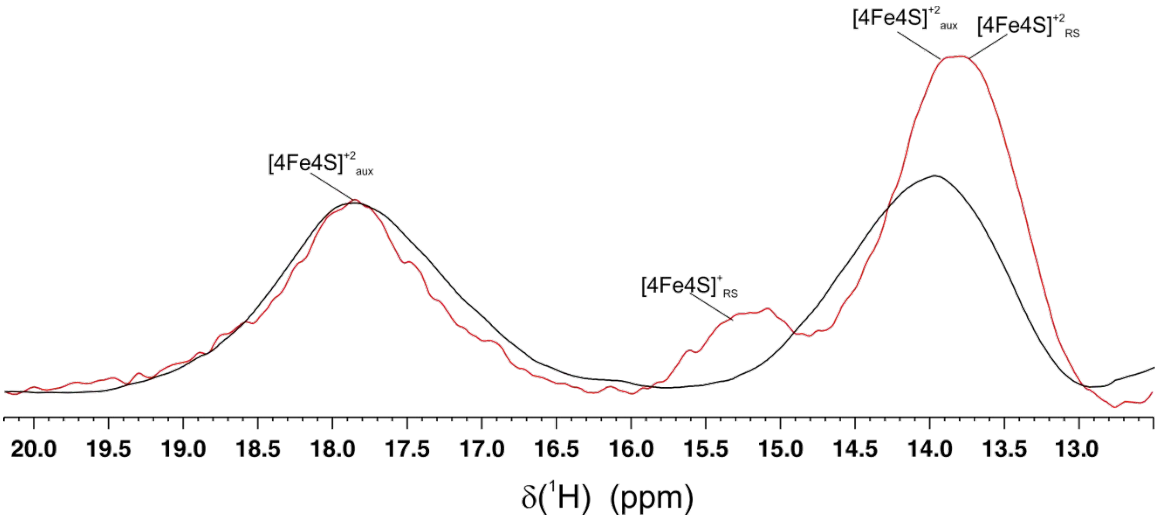

Figure S1. Al LIAS binds a $[4Fe\text{-}4S]^{2+}$ cluster at the FeS_{aux} site. Paramagnetic 1D ^{1}H NMR spectra of wild-type Al LIAS expressed with no addition of $FeCl_3$ at the time of induction (black) and wild-type Al LIAS expressed with the addition of 125 μM $FeCl_3$ at the time of induction (red). In the black spectrum, the signal at 17.9 ppm, which arises from a Hβ proton of a ligand bound to $[4Fe4S]_{aux}$ cluster,[3] has essentially the same intensity of the signal at 13.9 ppm, which arises from three protons, i.e. one due to a cluster ligand bound to the $[4Fe4S]_{aux}$ cluster and the other two due to cluster ligands bound to the $[4Fe4S]_{RS}$ cluster.[3] Moreover, the signal at 15.4 ppm, which arises from a Hβ proton of a ligand bound to the reduced form of the $[4Fe4S]^{+}_{RS}$ cluster,[3] is not observed in the black NMR spectrum. Overall, these results indicate that Al LIAS expressed with no addition of $FeCl_3$ at the time of induction mostly contains a [4Fe-4S] cluster bound at the FeS_{aux} site. The slow exchange kinetics on the NMR time scale here observed between oxidized and reduced cluster forms is in agreement with what already reported for other [4Fe-4S] proteins.[11]

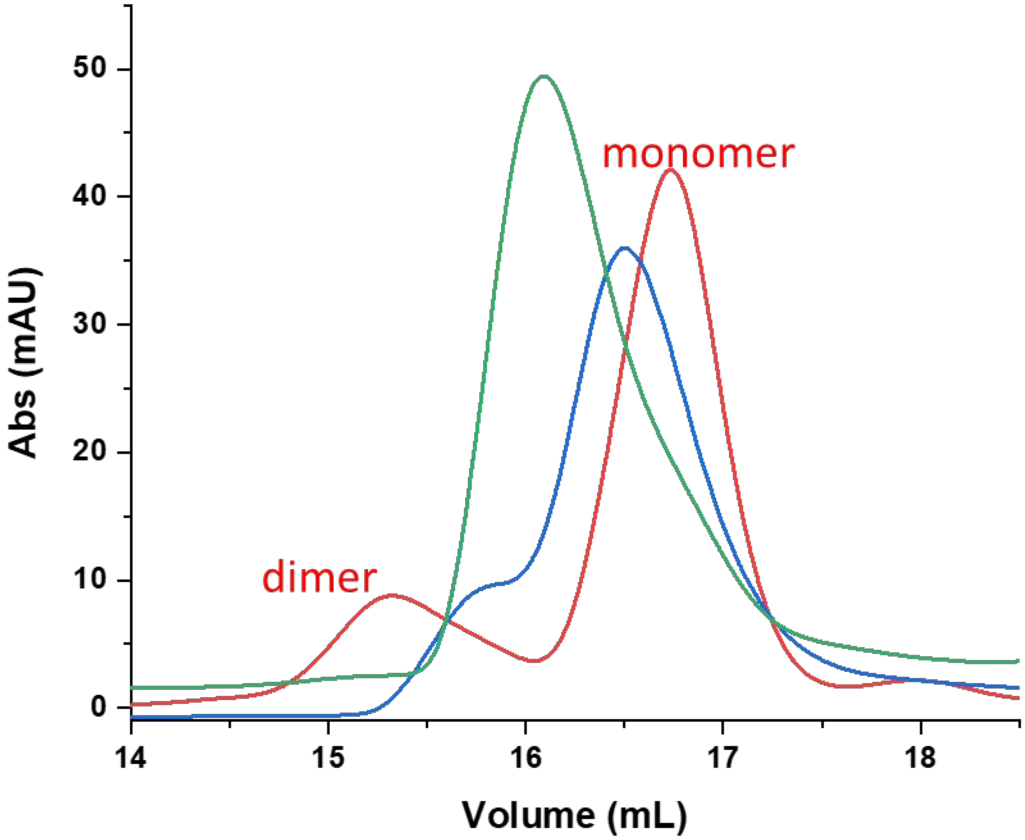

Figure S2. Analytical gel filtration of the heterodimeric complex formed by apo NFU1 and AI LIAS. The chromatograms of a 1:1 apo NFU1-AI LIAS mixture (green), AI LIAS (blue), apo NFU1 (red) are shown. The asymmetry to the right of the main peak (containing both proteins as detected by SDS-PAGE) in the apo NFU1-AI LIAS mixture indicates that a low percentage of free monomeric AI LIAS and apo NFU1 is present, supporting that a low portion of the complex dissociates in the experimental conditions used in the gel filtration. This suggests that the dissociation constant of the complex is in the low μM range. Taking into account the concentration of the two proteins in the NMR sample containing the 1:1 mixture here gel-filtrated, an upper limit of the dissociation constant of ~80 μM for the apo NFU1-AI LIAS complex can be estimated.

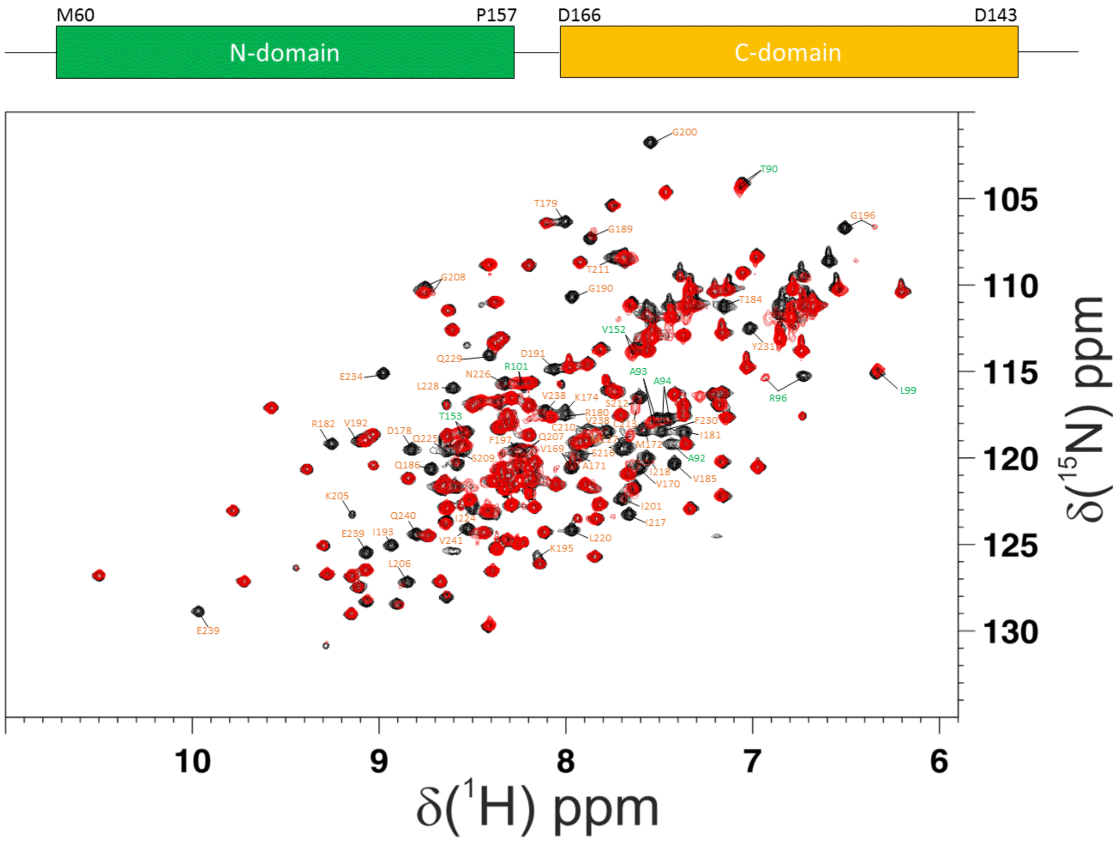

Figure S3. Complex formation between apo NFU1 and Al LIAS via the C-terminal domain of NFU1. Overlay of ^{1}H-^{15}N HSQC NMR maps of ^{15}N-labelled apo NFU1 (black) and the 1:1 ^{15}N-labelled apo NFU1-unlabelled AI LIAS mixture (red). Chemical shift assignment of the residues of the C- and N-domains affected by complex formation are shown according to the color code of the two domains depicted on the top of the figure. The majority of the residues display an intermediate exchange regime on the NMR time scale and belong to the C-domain of NFU1. Only a few residues of the N-domain significantly change their chemical shifts (they belong to the short helix formed by residues 90-96 and surrounding residues). The same effects were observed to occur in complex formation between ISCA1 and apo NFU1 as it was already deeply characterized and discussed in [2]. Among these residues, Arg 96 largely change its proton chemical shift in a slow exchange regime on the NMR time scale, thus allowing us to monitor that complete complex formation was reached at ~1:1 protein-protein ratio.

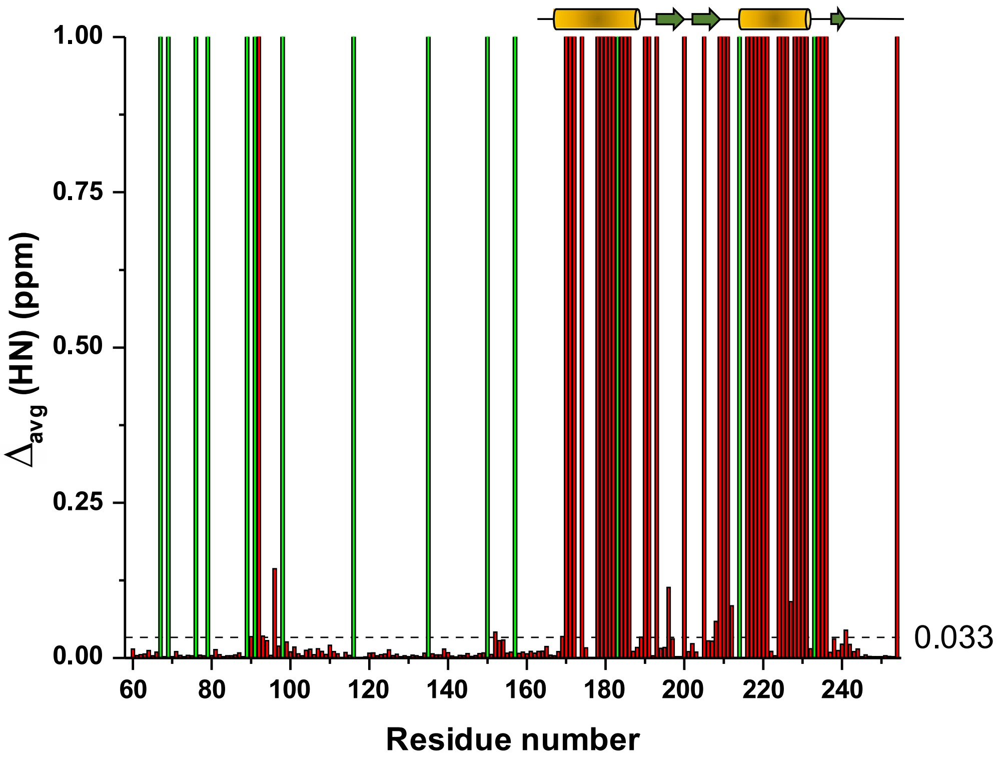

Figure S4. Apo NFU1 interacts with Al LIAS via its C-terminal domain. Backbone weighted average chemical shift differences Δ_{avg}(HN) (i.e., $(((\Delta H)^2 + (\Delta N/5)^2)/2)^{1/2}$) (red bars), between apo NFU1 and the 1:1 ^{15}N-labelled apo NFU1-unlabelled Al LIAS mixture. The indicated threshold values (obtained by averaging Δ_{avg}(HN) values plus 1σ) were used to define meaningful chemical shift differences. The green bars represent proline residues. The secondary structure elements of the C-domain of apo NFU1 are shown on the top. Only a few residues in the 90-96 region of the N-terminal domain showed meaningful chemical shift changes. These residues are the same as those affected in the apo ISCA1-NFU1 complex formation, supporting the previously proposed model:[1-2] these residues are located at an interacting region between the N- and C-domains of apo NFU1 and complex formation (for both ISCA1-NFU1 and Al LIAS-NFU1 complexes) induces changes in this intra-domain interaction.

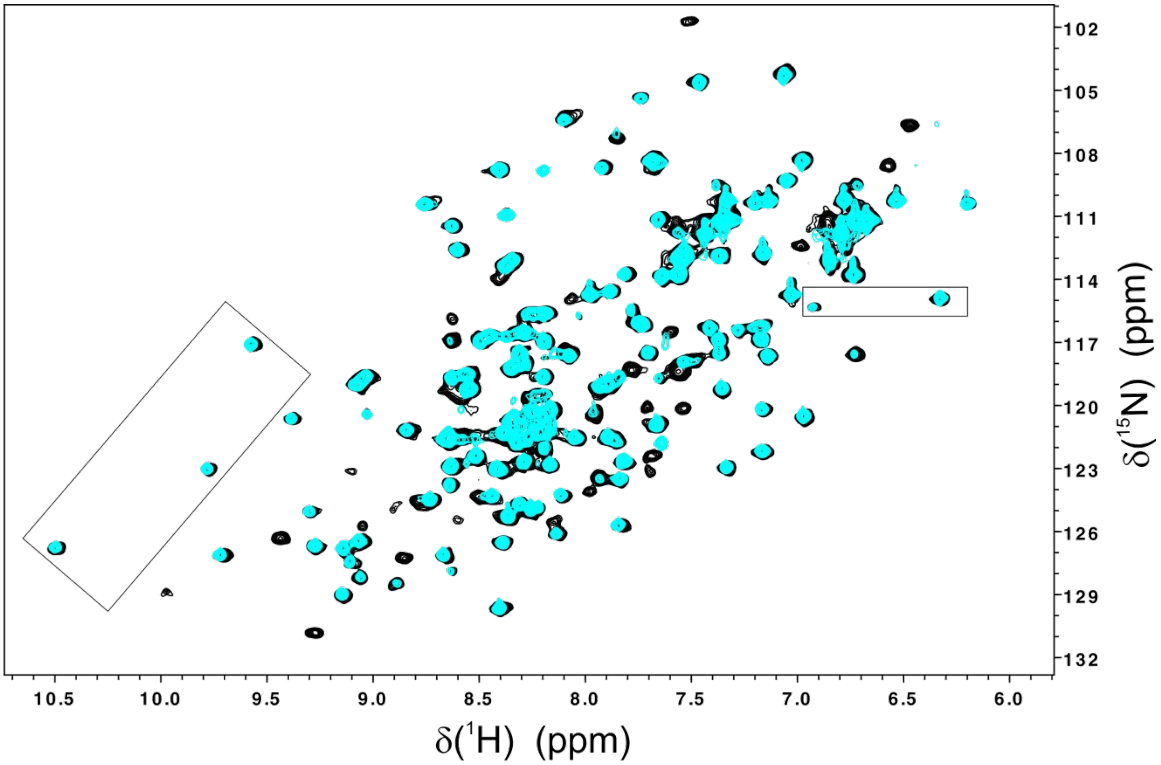

Figure S5. Comparison of the apo ISCA1-NFU1 and apo NFU1-Al LIAS complexes by NMR. Overlay of ^{1}H-^{15}N HSQC NMR maps of ^{15}N-labelled apo NFU1 complexed with ISCA1 (black) and with Al LIAS (cyan). This comparison clearly shows that the HSQC maps of the two complexes are different and thus they can be exploited to monitor the conversion between them. Indeed, in Figure 3A of the main text, the HSQC map of the 1:1 mixture between apo NFU1-ISCA1 complex and Al LIAS does not match with that of the isolated apo NFU1-ISCA1, while, in Figure 3B of the main text, the HSQC map of the 1:1 mixture between apo NFU1-ISCA1 complex and Al LIAS is well superimposable with that of the apo NFU1-Al LIAS complex, indicating that, in the final mixture, the apo NFU1-Al LIAS complex is formed as prevalent species. The black boxes identify some signals of NFU1 whose chemical shifts is not affected by complex formation of NFU1 with both Al LIAS and ISCA1. Thus, they are not affected by the conversion observed to occur between the two apo NFU1-ISCA1 and apo NFU1-Al LIAS complexes. On this basis, they are appropriate to be used to exclusively monitor cluster release from the NFU1-ISCA1 complex to Al LIAS (see Figure S6).

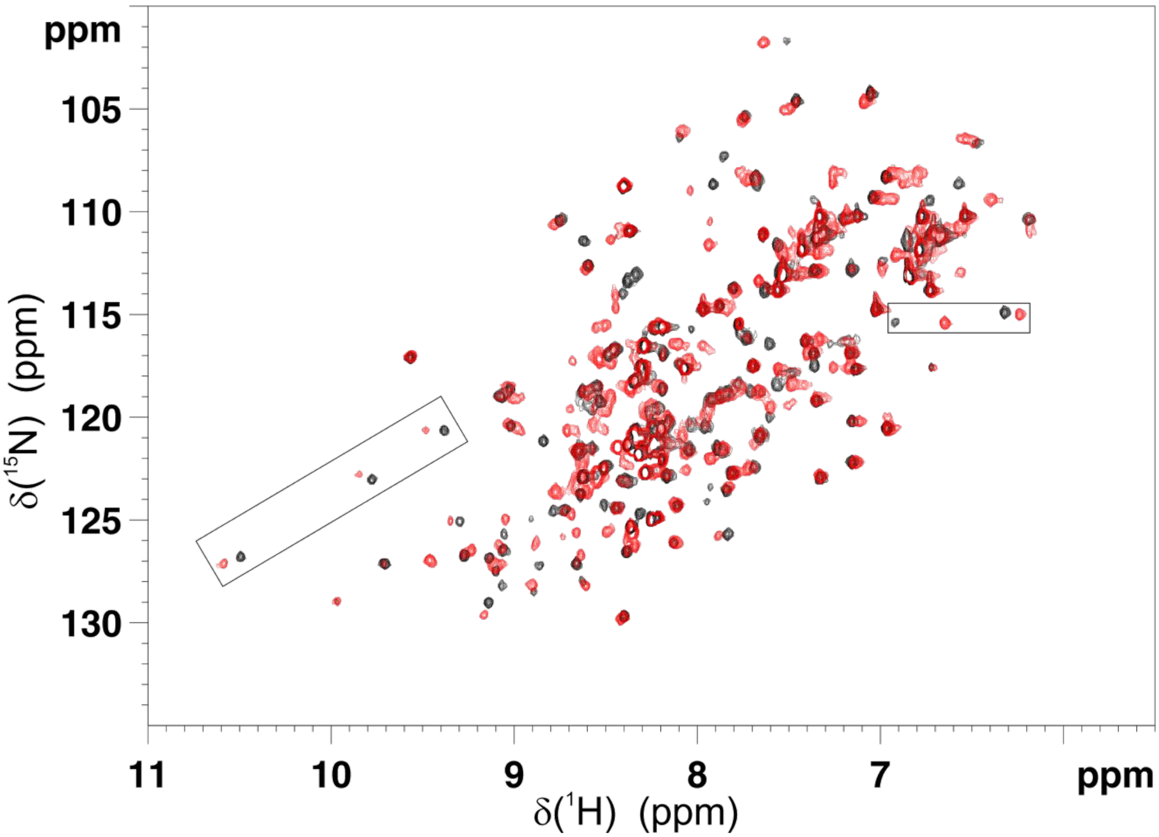

Figure S6. Comparison of NMR spectra of apo and [4Fe-4S]$^{2+}$ ISCA1-NFU1. Overlay of ^{1}H-^{15}N HSQC NMR maps of ^{15}N-labelled apo (black) and [4Fe-4S]$^{2+}$ NFU1 complexed with ISCA1 (red). The two boxes identify the signals affected by [4Fe-4S]$^{2+}$ cluster binding to the complex, but not affected by protein interaction of NFU1 with both ISCA1 and AI LIAS proteins (Figure S5), and are thus shown in Figure 4 of the main text to monitor cluster transfer from [4Fe-4S]$^{2+}$ ISCA1-NFU1 to AI LIAS.

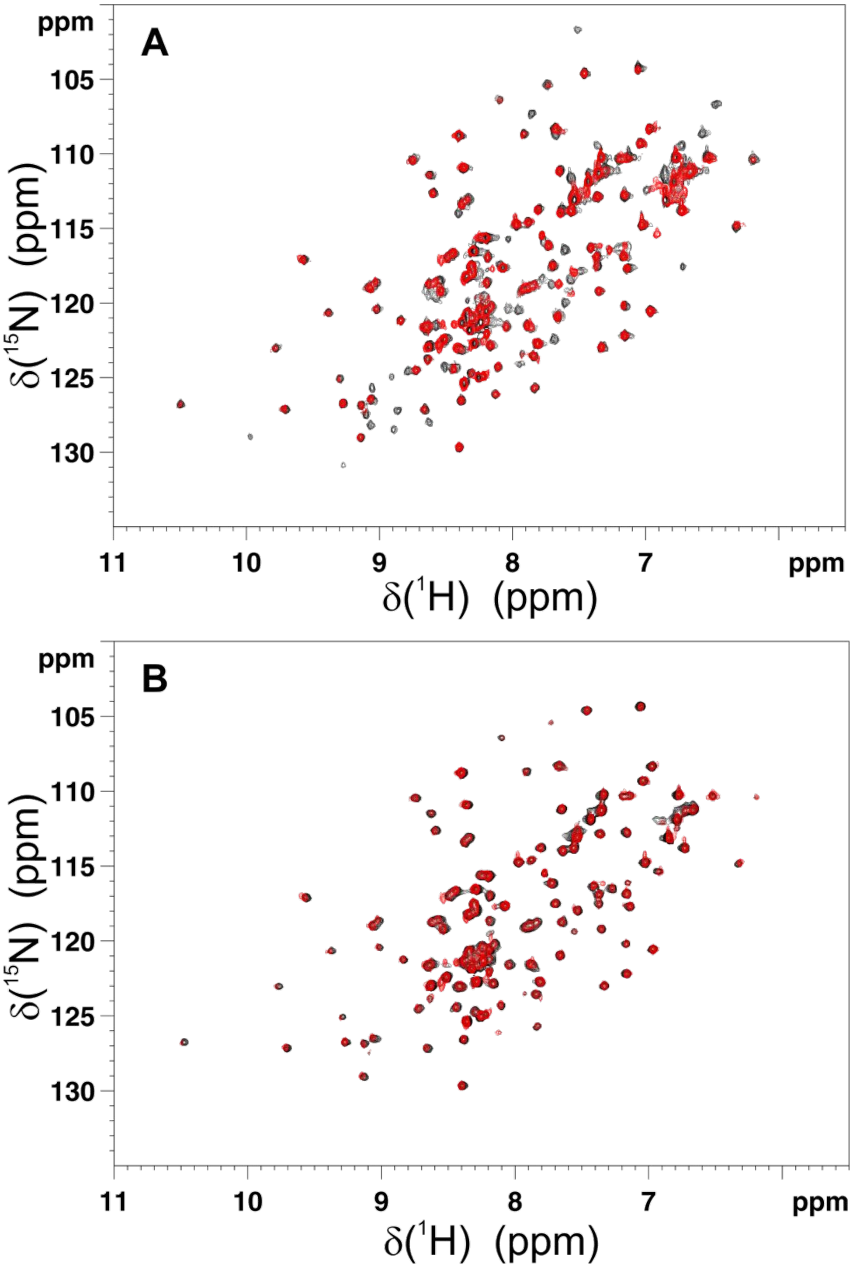

Figure S7. Cluster transfer from $[4Fe\text{-}4S]^{2+}$ ISCA1-NFU1 complex to Al LIAS to form apo NFU1-$[4Fe\text{-}4S]_2$ LIAS complex. (**A**) Overlay of ^{1}H-^{15}N HSQC NMR maps of ^{15}N-labelled apo NFU1 complexed with ISCA1 (black) and a 1:1 mixture of $[4Fe\text{-}4S]^{2+}$ ISCA1-^{15}N-labelled NFU1 and unlabelled Al LIAS (red). (**B**) Overlay of ^{1}H-^{15}N HSQC NMR maps of the apo ^{15}N labelled NFU1-Al LIAS complex (obtained by mixing apo ^{15}N labelled NFU1-ISCA1 complex with Al LIAS) (black) and a 1:1 mixture of $[4Fe\text{-}4S]^{2+}$ ISCA1-^{15}N-labelled NFU1 and unlabelled Al LIAS (red).

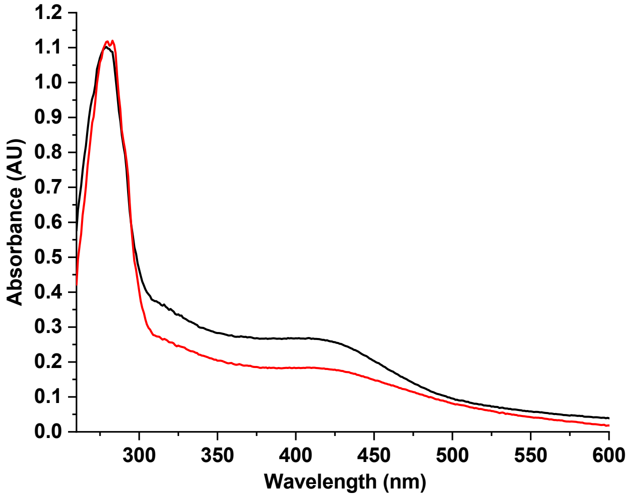

Figure S8. Cluster transfer from $[4Fe\text{-}4S]^{2+}$ ISCA1-NFU1 complex to AI LIAS by UV-visible spectroscopy. UV-visible spectra of C106/C111/C117A AI LIAS (red) and a 1:1 mixture of $[4Fe\text{-}4S]^{2+}$ ISCA1-NFU1 and C106/C111/C117A AI LIAS (black). Both spectra comprise a broad peak centered at 400 nm that is characteristic of $[4Fe\text{-}4S]^{2+}$ clusters.

References

1. Nasta, V.; Suraci, D.; Gourdoupis, S.; Ciofi-Baffoni, S.; Banci, L., A pathway for assembling [4Fe-4S]$^{2+}$ clusters in mitochondrial iron-sulfur protein biogenesis. *FEBS J* **2020,** *287*, 2312-2327.

2. Suraci, D.; Saudino, G.; Nasta, V.; Ciofi-Baffoni, S.; Banci, L., ISCA1 Orchestrates ISCA2 and NFU1 in the Maturation of Human Mitochondrial [4Fe-4S] Proteins. *J Mol Biol* **2021,** *433*, 166924.

3. Camponeschi, F.; Muzzioli, R.; Ciofi-Baffoni, S.; Piccioli, M.; Banci, L., Paramagnetic (1)H NMR Spectroscopy to Investigate the Catalytic Mechanism of Radical S-Adenosylmethionine Enzymes. *J Mol Biol* **2019,** *431*, 4514-4522.

4. Cicchillo, R. M.; Lee, K. H.; Baleanu-Gogonea, C.; Nesbitt, N. M.; Krebs, C.; Booker, S. J., Escherichia coli lipoyl synthase binds two distinct [4Fe-4S] clusters per polypeptide. *Biochemistry* **2004,** *43*, 11770-81.

5. Hendricks, A. L.; Wachnowsky, C.; Fries, B.; Fidai, I.; Cowan, J. A., Characterization and Reconstitution of Human Lipoyl Synthase (LIAS) Supports ISCA2 and ISCU as Primary Cluster Donors and an Ordered Mechanism of Cluster Assembly. *Int J Mol Sci* **2021,** *22*, 1598.

6. Banci, L.; Bertini, I.; Ciofi-Baffoni, S.; Boscaro, F.; Chatzi, A.; Mikolajczyk, M.; Tokatlidis, K.; Winkelmann, J., Anamorsin is a 2Fe2S cluster-containing substrate of the Mia40-dependent mitochondrial protein trapping machinery. *Chem. Biol* **2011,** *18*, 794-804.

7. Keller, R., *The Computer Aided Resonance Assignment Tutorial*. CANTINA Verlag: Goldau, 2004.

8. Williamson, M. P., Using chemical shift perturbation to characterise ligand binding. *Prog. Nucl. Magn Reson. Spectrosc* **2013,** *73*, 1-16.

9. Cai, K.; Liu, G.; Frederick, R. O.; Xiao, R.; Montelione, G. T.; Markley, J. L., Structural/Functional Properties of Human NFU1, an Intermediate [4Fe-4S] Carrier in Human Mitochondrial Iron-Sulfur Cluster Biogenesis. *Structure* **2016,** *24*, 2080-2091.

10. Inubushi, T.; Becker, E. D., Efficient detection of paramagnetically shifted NMR resonances by optimizing the WEFT pulse sequence. *J. Magn. Reson* **1983,** *51*, 128-133.

11. Bertini, I.; Gaudemer, A.; Luchinat, C.; Piccioli, M., Electron self-exchange in HiPIPs. A characterization of HiPIP I from *Ectothiorhodospira vacuolata*. *Biochemistry* **1993,** *32*, 12887-12893.
